# Candida Tropicalis Cholangitis in a Patient Without Underlying Malignancy

**DOI:** 10.7759/cureus.1867

**Published:** 2017-11-21

**Authors:** Arjun Saradna, Shyam Shankar, Benhoor Shamian, Yizhak Kupfer

**Affiliations:** 1 Internal Medicine, Maimonides Medical Center; 2 Critical Care, Maimonides Medical Center

**Keywords:** candida tropicalis, cholangitis, malignancy, critical illness, candida, gall bladder, septic shock, candida albicans, icu, intensive care unit

## Abstract

Candida tropicalis is a rare cause of acute cholangitis, predominantly seen in patients with underlying hematological malignancies. Here, we describe a case of acute cholangitis caused by mixed organisms (Candida tropicalis, Candida albicans, and Enterococcus durans) without a known risk factor.

## Introduction

Acute cholangitis is a clinical syndrome characterized by fever, jaundice, and abdominal pain that develops as a result of stasis and infection in the biliary tract. It is primarily caused by a bacterial infection in a patient with biliary obstruction. The organisms typically ascend from the duodenum into the biliary tract; other uncommon mechanisms include hematogenous spread from the portal vein. 

Candida tropicalis is a rare cause of acute cholangitis, predominantly seen in patients with underlying hematological malignancies or other forms of immunosuppression [1]. Here, we describe a case of acute cholangitis caused by mixed organisms (Candida tropicalis, Candida albicans, and Enterococcus durans) without known risk factors.

## Case presentation

A 78-year-old Chinese female was brought to the emergency department by her daughter for an unwitnessed fall at home. Upon presentation, the patient was minimally responsive on examination with grimacing on abdominal palpation. Initial vitals showed evidence of fever to 102 degrees Fahrenheit, hypotension with a blood pressure of 86/42 millimeters of mercury (mmHg), and normal oxyhemoglobin saturation on room air. Initial laboratory evaluation revealed leukocytosis with neutrophilic predominance. Liver function tests demonstrated total and direct hyperbilirubinemia with total bilirubin of 9.3 milligrams per deciliter (mg/dl) and direct bilirubin of 6.1 mg/dl, elevated transaminase levels with an aspartate transaminase (AST) of 123 international units per milliliters (IU/ml), and alanine transaminase (ALT) of 40 IU/ml. Her alkaline phosphatase was elevated to 419 IU/ml. Urinalysis showed orange urine with no hemoglobin, a large amount of bilirubin, positive nitrite, 4 mg/dl urobilinogen, small leukocyte esterase, few bacteria, and 2-5 white blood cells per high-powered field (WBC/HPF). 

**Figure 1:**
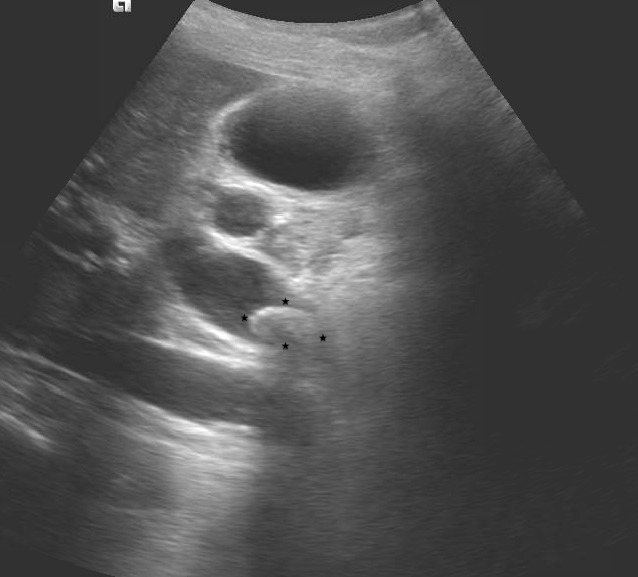
Ultrasound showing significant proximal common bile duct dilatation measuring 2.1 cm A stone within the proximal to mid-common bile duct measuring 8.9 mm in short axis by 15.1 mm in long axis is visualized.

**Figure 2:**
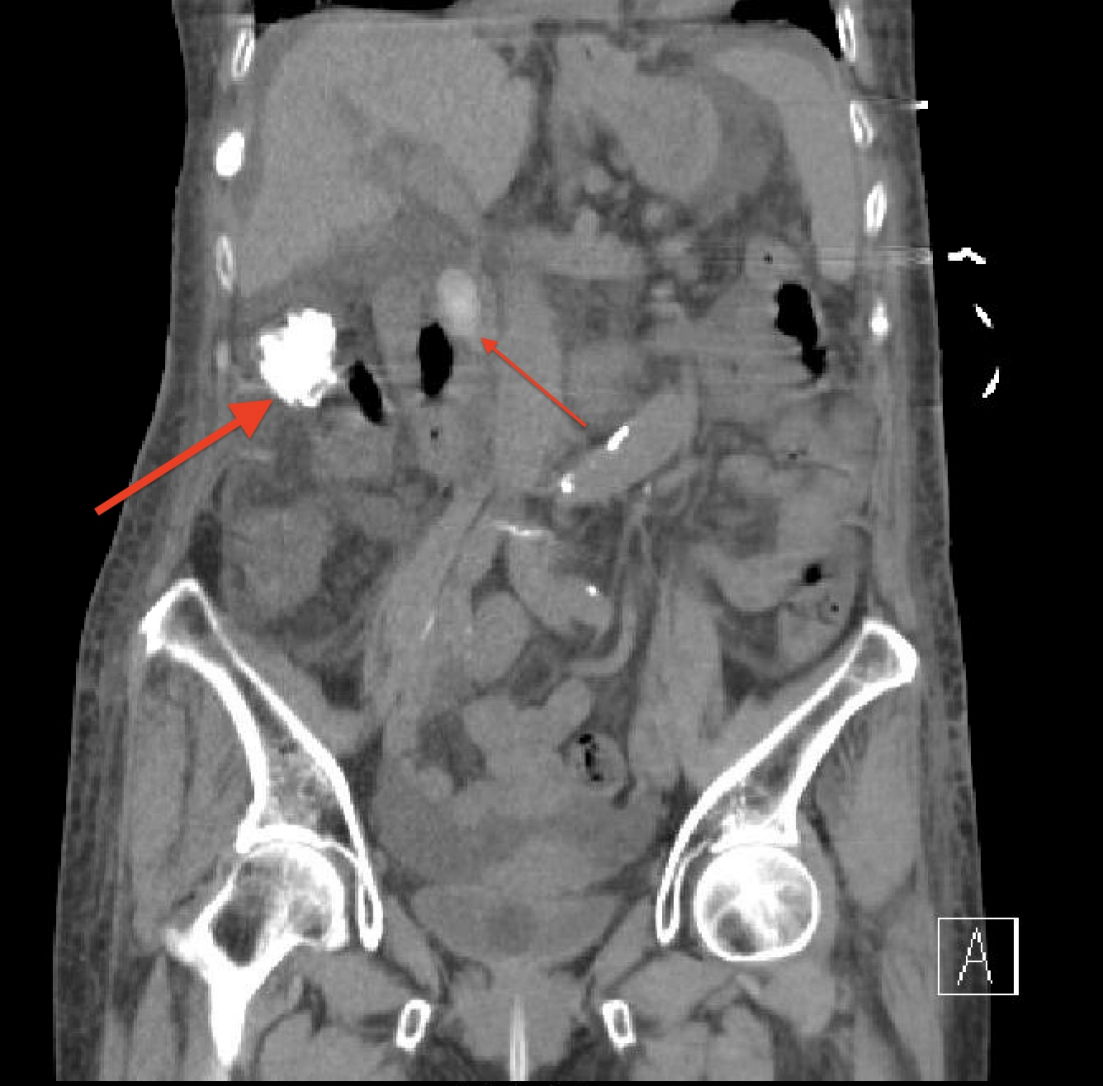
Computed tomography coronal image showing proximal/mid-common bile duct stone (thin arrow) Additional layering stones (thick arrow) in the gallbladder are also visualized. Intra and extra-hepatic ductal dilatation is seen.

Given the findings of abdominal tenderness and elevated transaminase levels, an ultrasound of the abdomen was obtained which revealed mild to moderate intrahepatic bile duct dilatation and a significant proximal common bile duct (CBD) dilatation measuring 2.1 centimeters (cm). A proximal CBD stone was visualized with mild distention of the gallbladder (Figure 1). A computed tomography scan (CT) of the chest, abdomen, and pelvis without contrast was obtained which showed evidence of choledocholithiasis (Figure 2). A CT of the head ruled out an acute bleed and any intracranial masses. 

The patient continued to be hypotensive after adequate fluid resuscitation per the sepsis protocol, and a norepinephrine drip was started. Blood cultures were sent and empiric antibiotic treatment for septic shock secondary to cholangitis with vancomycin, cefepime, and metronidazole was started. Acid suppression with intravenous pantoprazole was initiated. The patient was transferred to the medical intensive care unit (ICU) for intensive monitoring and hemodynamic support. The family refused any surgical interventions after a discussion of the abdominal ultrasound findings. Considering the patient’s clinical condition, a decision was made to place an interventional radiology-guided percutaneous biliary drain distal to the obstruction. Cultures were sent from the percutaneous drain. Improvement in the temperature curve, a reduction of the leucocyte count, and a reduction in the pressor requirement were noted after placement of the percutaneous drain. Blood cultures grew pan-sensitive Escherichia coli so the antibiotics were changed to address the sensitivities of the organism. The patient was tapered off the norepinephrine drip one day post-drain placement. On Day 4 of her admission, the patient had an episode of oxyhemoglobin desaturation and was placed on high-flow oxygen to keep the oxygen saturation above 95%. Oxyhemoglobin saturation fell below 90% again on high-flow and the patient had to be emergently intubated. Broad spectrum antibiotics were started. The mean arterial pressure was less than 60 mmHg so a norepinephrine drip was reinitiated. Laboratory results at that time revealed a WBC count of 19 thousand per microliter (K/uL) from a count of 9 K/uL drawn 24 hours previously. The family was notified of the event and wished for the patient to be provided with comfort care with a plan for palliative extubation to be performed by the pain and palliative team on the next day. The patient was palliatively extubated and she passed away. 

One day after patient’s death, cultures sent from the bile fluid grew 3+ Candida tropicalis, Enterococcus durans, and 1+ Candida albicans.

## Discussion

The classic presentation of acute cholangitis is fever, abdominal pain, and jaundice (Charcot's triad), though only 50% - 75% of patients with acute cholangitis have all three findings [2]. Confusion and hypotension can occur in patients with suppurative cholangitis, producing Reynold’s pentad, which is associated with significant morbidity and mortality [3]. If septic shock develops, multi-organ failure may be seen. Hypotension may be the only presenting symptom in elderly patients or those on glucocorticoids. Our patient presented with an altered mental status, abdominal tenderness, hypotension, elevated transaminase and bilirubin levels, and fever, which concords with most of the features of Reynold’s pentad. 

Acute cholangitis is caused primarily by a bacterial infection in patients with biliary obstruction. Bile taken from patients without obstruction is sterile or nearly sterile. By comparison, approximately 70% of all patients with gallstones have evidence of bacteria in the bile. Patients with common bile duct stones have a higher probability of bile culture positivity than those with gallstones in the gallbladder or cystic duct [4].

A culture of bile in patients with ductal stones and blocked biliary stents are positive in over 90% of cases of acute cholangitis, yielding mostly a mixed growth of gram-negative and gram-positive bacteria. The most common gram-positive organism is Enterococcus (10%). Candida in bile cultures is usually considered to be a colonization and not treated unless known risk factors are present, such as underlying malignancy, history of prolonged antibiotic use, immunosuppression, or biliary stents. There are three possible pathways for the spread of Candida into the biliary tract, which include ascending spread via the duodenum, hematogenous spread from fungemia and sepsis, and via instrumentation, such as endoscopic retrograde cholangiopancreatography (ERCP)/stenting. Our patient did not have fungemia or instrumentation/stents, so the most probable mechanism was ascending spread from the duodenum. 

We describe a case of acute cholangitis who decompensated on optimal antibiotic therapy with bile cultures later growing Candidatropicalis and Candida albicans, in addition to Enterococcus durans. The patient was initially treated for two days with broad-spectrum antibiotics after which blood cultures grew pan-sensitive E. coli and the antibiotic spectrum was narrowed to cover the sensitivities of the E. coli. On Day 4, the patient’s condition worsened and vancomycin, cefepime, and metronidazole were reinitiated to cover a broader spectrum of microorganisms. Enterococcus in the bile cultures was also pan-sensitive so was covered in both instances. Throughout the course, optimal antibiotic coverage was provided except for Candida.

Cholangitis secondary to Candida tropicalis has mostly been defined in patients with underlying malignancy. Our case had a negative malignancy workup, including computed tomography scans of the head/chest/abdomen/pelvis, no history of a previous ERCP, and no history of recent long-term antibiotic administration. Blood cultures were negative for Candida, which is concordant with various studies which reported the sensitivity of blood cultures to detect Candida to be 50-75% or lower [5].

## Conclusions

We describe a case of Candida tropicalis cholangitis in a patient without underlying malignancy or other risk factors. Hence, biliary candidiasis should be considered as a cause of cholangitis in immunocompetent patients, especially in the ICU setting, and Candida in bile cultures should not be regarded as a colonizer but should be correlated with the clinical setting. Consideration should be given to early initiation of empiric antifungal therapy in patients with cholangitis if clinical improvement doesn't occur with optimal antibiotic therapy. Candidemia, as discussed, is not frequently present in patients with biliary candidiasis.

This case also highlights the importance of advance directives in elderly patients and the importance to have a care plan congruent with the patient's wishes. 

## References

[1] Ballal M, Chakraborty R, Bhandary S, Kumar PS (2013). Candida tropicalis in a case of cholangiocarcinoma with cholangitis at a tertiary care hospital in Manipal. Med Mycol Case Rep.

[2] Saik RP, Greenburg AG, Farris JM, Peskin GW (1975). Spectrum of cholangitis. Am J Surg.

[3] DenBesten L, Doty JE (1981). Pathogenesis and management of choledocholithiasis. Surg Clin North Am.

[4] Csendes A, Becerra M, Burdiles P (1994). Bacteriological studies of bile from the gallbladder in patients with carcinoma of the gallbladder, cholelithiasis, common bile duct stones and no gallstones disease. Eur J Surg.

[5] Lenz P, Eckelskemper F, Erichsen T (2014). Prospective observational multicenter study to define a diagnostic algorithm for biliary candidiasis. World J Gastroenterol.

